# Head Injury Prevalence in a Population of Injured Patients Seeking Care in Ghana, West Africa

**DOI:** 10.3389/fneur.2022.917294

**Published:** 2022-06-20

**Authors:** Frank Baiden, Martina Anto-Ocrah, George Adjei, Stephaney Gyaase, Jacob Abebrese, Damien Punguyire, Seth Owusu-Agyei, Rachel T. Moresky

**Affiliations:** ^1^Department of Epidemiology and Biostatistics, School of Public Health, University of Health and Allied Sciences, Hohoe, Ghana; ^2^Department of Medicine, Division of Internal Medicine, University of Pittsburgh School of Medicine, Pittsburgh, PA, United States; ^3^Department of Community Medicine, School of Medical Sciences, University of Cape Coast, Cape Coast, Ghana; ^4^Kintampo Health Research Centre, Ghana Health Service, Kintampo, Ghana; ^5^Institutional Care Division, Ghana Health Service, Accra, Ghana; ^6^Upper West Regional Health Directorate, Ghana Health Service, Wa, Ghana; ^7^Institute of Health Research, University of Health and Allied Sciences, Ho, Ghana; ^8^SidHARTe-Strengthening Emergency Systems Program, Heilbrunn Department of Population and Family Health Columbia University, Mailman School of Public Health, New York, NY, United States; ^9^Department of Emergency Medicine Columbia University, Vagelos College of Physicians and Surgeons, New York, NY, United States

**Keywords:** head injury, traumatic brain injury, Ghana, injury registry, Africa, emergency medicine

## Abstract

**Background/Significance:**

Much of the literature on head injury (HI) prevalence comes from high-income countries (HICs), despite the disproportionate burden of injuries in low to middle-income countries (LMICs). This study evaluated the HI prevalence in the Kintampo Injury Registry, a collaborative effort between Kintampo Health Research Centre (KHRC) in Ghana and the sidHARTe Program at Columbia University Mailman School of Public Health. In our first aim, we characterize the HI prevalence in the registry. In aim 2, we examine if there are any sex (male/female) differences in head injury outcomes in these populations for points of potential intervention.

**Methods:**

Secondary analysis of data from the Kintampo Injury Registry which had 7,148 registered patients collected during January 2013 to January 2015. The definition of a case was adopted to ensure consistency with the International Statistical Classification of Diseases and Related Health Problems, revision 10 (ICD-10). A 3-page questionnaire was used to collect data from injured patients to include in the registry. The questions were designed to be consistent with the World Health Organization (WHO) guidelines on injury surveillance and were adapted from the questionnaire used in a pilot, multi-country injury study undertaken in other parts of Africa. The questionnaire collected information on the anatomic site of injury (e.g., head), mechanism of injury (e.g., road traffic injuries, interpersonal injuries (including domestic violence), falls, drowning, etc.), severity and circumstances of the injury, as well as precipitating factors, such as alcohol and drug use. The questionnaire consisted mainly of close-ended questions and was designed for efficient data entry. For the secondary data analyses for this manuscript, we only included those with “1st visit following injury” and excluded all transfers and follow-up visits (*n* = 834). We then dichotomized the remaining 6,314 patients to head injured and non-head injured patients based on responses to the variable “Nature of injury =Head Injury”. We used chi-square and Fisher's exact tests with *p* < 0.05 as cut-off for statistical significance. Logistic regression estimates were used for effect estimates.

**Results:**

Of the 6,314 patients, there were 208 (3.3%) head-injured patients and 6,106 (96.7%) patients without head injury. Head-injured patients tended to be older (Mean age: 28.9 +/-13.7; vs. 26.1 +/- 15.8; *p* = 0.004). Seven in 10 head injured patients sustained their injuries *via* transport/road traffic accidents, and head-injured patients had 13 times the odds of mortality compared with those without head injuries (OR: 13.3; 95% CI: 8.05, 22.0; *p* < 0.0001) even though over half of them had mild or moderate injury severity scores (*p* < 0.001). Evaluation of sex differences amongst the head-injured showed that in age-adjusted logistic regression models, males had 1.4 times greater odds of being head injured (OR: 1.4; 95% CI: 1.04, 2.00; *p* = 0.03) and over twice the risk of mortality (OR: 2.7; 95% CI: 0.74, 10.00; *p* = 0.13) compared to females.

**Conclusion:**

In these analyses, HI was associated with a higher risk of mortality, particularly amongst injured males; most of whom were injured in transport/road-traffic-related accidents. This study provides an impetus for shaping policy around head injury prevention in LMICs like Ghana.

## Introduction

Head injuries (HI) represent more than 50% of all injuries and are one of the leading causes of death and disability globally (1, 2). They not only result in many years of productive life lost, but also impose immense economic costs for individuals, families, and societies (3). Commonly referred to as brain injury or traumatic brain injury (TBI) (4), head injuries can be presented as mild i.e., a bump, bruise (contusion), cut on the head, or moderate to severe. According to the most recent reports from the Global Burden of Diseases Study, from 1990 to 2016, the age-standardized incidence and prevalence of TBI increased by 3.6 and 8.4%, respectively, and there were 27.08 million new cases of traumatic HI in 2016 alone (3); making the injury more common than breast cancer, spinal cord injury, HIV/AIDS, and multiple sclerosis (MS) combined. Epidemiological studies estimate that 57 million people worldwide live with the neurological sequelae of HI, of which 10 million require hospital-based care (5). However, much of these estimates are based on data from predominantly global north populations with little to no representation of low and middle-income countries (LMICs), particularly those in the African region (1, 6–8). To move the public health needle forward and further inform clinical care, scientific publications should be more inclusive of diverse racial, ethnic, cultural and social groups globally. More research from the African region is needed to better understand the global variations in head injury prevalence and develop comprehensive and inclusive policies to address global disparities in head injury morbidity and mortality.

Investigators across parts of the African region have been taking steps in recent years to develop hospital-based trauma registries in several sub-Saharan African (SSA) countries to increase the generalizability and representation of African countries in epidemiologic reports (9–14). Few, however, have provided assessments of the head injury prevalence across these populations. Injuries have long been recognized as an important cause of morbidity and mortality in the West African country of Ghana (15–17). However, little exists in the literature on the demographic and injury-related attributes of those who sustain head injuries.

Heeding the call to “Advance the Representation of Minoritized Groups and Social Determinants of Health in Brain Injury Research,” we determine the head injury prevalence in a population of injured patients presenting for care in two Emergency Department facilities located in the middle-belt region of Ghana. We describe the demographic and injury-related attributes of the head-injured and secondly examine if there are any sex (male/female) differences in head injury outcomes in the population for points of intervention. To the best of our knowledge, this is the first epidemiological assessment of the burden of head injuries in the middle-belt region of the country, an area of the country that is often underrepresented in the literature. The overall goal of this endeavor is to advance the diversification of the scientific investigation of the global burden of head injuries by providing data from an underrepresented part of the world.

## Methods

### Setting

Ghana is a West African nation with a population of 30.8 million (18). Over the last three decades, Ghana's urban population has more than tripled, rising from 4 million to nearly 14 million people, and outpacing rural population growth (19). Ghana's per capita income as at 2017 was 1,641.49 USD, and the country is now classified among lower-middle-income countries (19).

### The Kintampo Injury Registry

**Figure 1:**
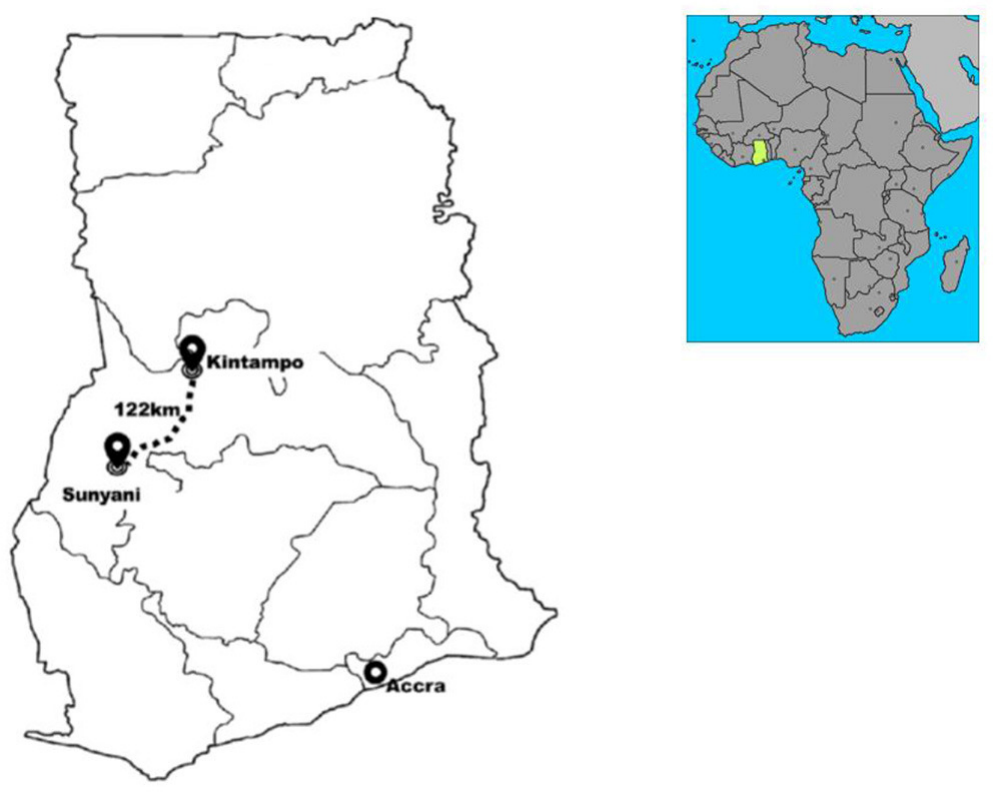
Map of Ghana showing the two study sites, Sunyani and Kintampo.

Data on injuries in Ghana are currently collected within the routine health management information system of various health facilities. This is done across all health facilities in the country, but in a non-standardized form. Therefore, the depth of inquiry into cases of injuries varies from one facility to another, making it nearly impossible to collate and analyze data across different facilities. It is against this background that the Kintampo Health Research Centre (KHRC), in collaboration with the sidHARTe–Strengthening Emergency Systems Program at Columbia University Mailman School of Public Health, initiated a pilot, facility-based injury registry in two hospitals in the Brong Ahafo Region of Ghana during the period from January 2013 to January 2015. The two facilities included in this initiative were the Brong Ahafo Regional Hospital in Sunyani (which serves a predominant urban patient population) and the Kintampo Municipal Hospital in Kintampo (which serves a predominantly sub-urban and rural patient population) (See map, Figure 1). The objective of this initiative was to gain experience in the establishment and maintenance of an injury registry in Ghana, analyze the data and make recommendations to inform the implementation of a national injury registry that would be more homogenous and accessible than the fragmented systems in place. The pilot project was initially intended to run for 12 months but was extended for an additional 12 months to be able to capture seasonal variations in injury trends over the data collection period.

### Case Definition

A case was classified as an injury and eligible to be entered into the registry when there was physical damage to a person resulting from sudden exposure to intolerable levels of energy. The injury could be in the form of bodily lesions or due to impairment of function resulting from a lack of one or more vital elements (i.e., air, water, heat), as in strangulation, drowning or freezing. The energy causing the injury could be mechanical (e.g., impact with a moving or stationary object, such as a surface, knife, or vehicle), radiant (e.g., a blinding light or a shock wave from an explosion), thermal (e.g., air or water that is too hot or too cold), electrical or chemical (e.g., a poison or an intoxicating or mind-altering substance such as alcohol or drugs). This definition was adopted to ensure consistency with definitions in Chapters XIX and XX of the International Statistical Classification of Diseases and Related Health Problems, revision 10 (ICD-10) (20). We included injuries of all severities, including head injury.

Informed consent was obtained from each injured person before inclusion in the registry. For children (<18 years), and adult patients (≤ 18 years) who arrived in an unconscious or severe state, consent was obtained from adult relatives. Ethical and administrative approval for the project was obtained from the Institutional Ethics Committee of KHRC, the Institutional Review Board at Columbia University and authorities of the participating health facilities.

### Instrument and Data Collection

A 3-page questionnaire was used to collect data from injured patients. The questions were designed to be consistent with the WHO guidelines on injury surveillance (20) and were adapted from the questionnaire used in a pilot, multi-country injury study undertaken in five African countries in 2006 (21). The questionnaire collected information on the anatomic site of injury (e.g., head), mechanism of injury [e.g., road traffic injuries, interpersonal injuries (including domestic violence), falls, drowning, etc.], severity and circumstances of the injury, as well as precipitating factors, such as alcohol and drug use. The questionnaire consisted mainly of closed-ended questions and was designed for efficient data entry.

The questionnaire was administered by trained personnel with a minimum qualification of an undergraduate degree (site coordinators) and trained regular health service staff of the two hospitals. These personnel were selected as site coordinators instead of health workers in order to reduce the cost of maintaining the registry. Staff was on hand 24 h a day, seven days a week, for recruitment and questionnaire administration. To ensure 24-h coverage for the capture and entry of cases, staff whose work related directly to the management of injuries were also trained to administer the questionnaire. Although the questionnaire was created in English, it was administered in the local language, mostly Twi.

While interviewers were encouraged to register cases as soon as patients arrived at the facility, detailed questionnaire administration was delayed until the patient had been stabilized. When an injured person was not in a position to be interviewed, a relative or another proxy who was present when the injury occurred was interviewed. After the patient had been stabilized and was able to interact, the information provided by the proxy was confirmed with the patient. When an injured person was admitted to the hospital, the registry was updated daily. Proxy interviews were conducted for those who had died at the time of the interview. No direct identifiers (e.g., names) were used on the data collection forms. Hospital identification numbers were used to follow up with patients. Each site was provided with a locked cabinet where completed forms were stored until the project coordinator collected them for data entry. The data obtained was kept strictly confidential and made available only to persons connected with the study.

### Analyses

For this paper, we conducted a retrospective, secondary data analyses of the 7,148 patients recruited and enrolled in the Kintampo Injury Registry over the 24 month recruitment period to identify and analyze the head injury data. We only included those with “1st visit following injury” and excluded all transfers and follow-up visits (n=834), leaving 6,314 patients to analyze. “Head injury” was classified as a “Yes” if “Nature of injury =Head Injury” was selected. We dichotomized the remaining study sample of 6,314 patients into head injured and non-head injured patients and compared the groups using chi-square and Fisher's exact tests, with *p* < 0.05 as the statistical cut-off for significance. Logistic regression estimates were used to determine crude and age-adjusted effect estimates comparing various outcomes within the head-injured subgroup.

## Results

Of the 6,314 patients, there were 208 (3.3%) head-injured patients and 6,106 (96.7%) patients without head injury (Table 1). Head injured patients tended to be older (Mean age: 28.9 +/-13.7; vs. 26.1 +/- 15.8; *p* = 0.004), with over half between the ages of 20 and 39. The proportion of injured male patients outnumbered that of females, and 3 in 4 head-injured patients (75.6%) were male (*p* = 0.0138) and had not attained any tertiary education (*p* = 0.0095).

**Table 1:** Demographic characteristics of head injury patients in the Kintampo Registry (*n* = 6,314).

**Variable**	**Head injured**	**Non-head injured**	**Total**	***p*-value**
	*n* =208 (3.3%)	*n* = 6,106 (96.7%)	*n* = 6,314	
**Age (years)**				
Mean (± standard deviation)	28.9 (±13.7)	26.1(±15.8)*	-	0.004
Median (interquartile range)	29 (20–36)	25 (15–35)*	-	-
**Age distribution (Years)** ***n*****, (%)**				
≤ 19	50 (24.0)	2,150 (35.2)	2,200 (34.9)	0.007
20–39	118 (56.7)	2,912 (47.7)	3,030 (48.0)	
40–59	34 (16.4)	825 (13.5)	859 (13.6)	
≥ 60	6 (2.9)	217 (3.6)	223 (3.5)	
Missing	0 (0.0%)	2 (0.03)	2 (0.03)	
**Sex**				
Male	157 (75.5)	4,113 (67.4)	4,270 (67.6)	0.0138
Female	51 (24.5)	1,993 (32.6)	2,044 (32.4)	
**Marital status**				
Single	114 (54.8)	3,472 (56.9)	3,586 (56.8)	0.66
Married	73 (35.1)	2,101 (34.4)	2,174 (34.4)	
Divorced/separated	5 (2.4)	75 (1.2)	80 (1.3)	
Widowed	2 (1.0)	52 (0.9)	54 (0.9)	
Not applicable	14 (6.7)	406 (6.7)	420 (6.7)	
**Religion**				
Christian	166 (79.8)	4,656 (76.3)	4,822 (76.4)	<0.001
Moslem	31 (14.9)	1,343 (22.0)	1,374 (21.8)	
Traditionalist	0 (0.0)	43 (0.7)	43 (0.7)	
Other	11 (5.3)	64 (1.1)	75 (1.2)	
**Employment status and occupation**				
Unemployed	32 (15.4)	582 (9.5)	614 (9.7)	0.0095
Student	48 (23.1)	2,091 (34.3)	2,139 (33.9)	
Farmer	37 (17.8)	1,107 (18.1)	1,144 (18.1)	
Business (wo)man	17 (8.2)	440 (7.2)	457 (7.2)	
Commercial driver	14 (6.7)	274 (4.5)	288 (4.6)	
Petty trader	14 (6.7)	341 (5.6)	355 (5.6)	
Other government worker	11 (5.3)	319 (5.2)	330 (5.2)	
Other	28 (13.1)	643 (10.5)	671 (10.6)	
Not applicable	7 (3.4)	309 (5.1)	316 (5.0)	
**Highest level of education**				
None	65 (31.3)	1,851 (30.3)	1,916 (30.4)	0.085
Primary (US Grades 1–6)	36 (17.3)	1,304 (21.4)	1,340 (21.2)	
JHS/SHS/Technical (US Grades 7–12)	89 (42.8)	2,267 (37.1)	2,356 (37.3)	
Tertiary (graduate/undergraduate levels)	14 (6.7)	510 (8.4)	524 (8.3)	
Post-Graduate	1 (0.5)	4 (0.07)	5 (0.1)	
Other	3 (1.4)	170 (2.8)	173 (2.7)	

*Missing 2 observations.

There were several statistically significant differences in injury attributes between the head-injured patients and those without head injuries (Table 2). Although most patients arrived *via* taxi or commercial vehicle, head injured patients were more likely to be transported *via* ambulance than non-head injured patients (8.7 vs. 3.8%; *p* < 0.001). A greater proportion was either dead on arrival or unconscious (*p* < 0.001) even though over half had mild or moderate injury severity scores (*p* < 0.001) based on the Kampala Trauma Score (KTS II), which has been deemed as effective as other scoring systems for predicting patient mortality in LMIC settings (22). Although 93% of head-injured patients (vs. 75% of those without head injuries) were discharged without consequence, a significantly greater proportion was either referred to higher-level facilities or succumbed to their injuries and died (*p* < 0.001). Head injured patients had 13 times the odds of mortality compared to those without head injuries (OR: 13.3; 95% CI: 8.05, 22.0; *p* < 0.0001) (data not shown).

**Table 2:** Injury characteristics of patients in the Kintampo Registry, stratified by head injury status (*n* = 6,314).

**Variable**	**Head injured**	**Non-head injured**	**Total**	***p*-value**
	*n* = 208 (3.3%)	*n* = 6,106 (96.7%)	*n* = 6,314	
**State of consciousness at arrival**			
Alive and Conscious	177 (85.1)	6,033 (98.8)	6,210 (98.4)	<0.001
Alive but unconscious	26 (12.5)	51 (0.84)	77 (1.2)	
Dead	5 (2.4)	22 (0.4)	27 (0.4)	
**Mode of arrival to facility**				
Taxi/commercial vehicle	170 (81.7)	5,235 (85.7)	5,405 (85.6)	
Ambulance	18 (8.7)	231 (3.8)	249 (3.9)	<0.001
Private vehicle	14 (6.7)	179 (2.9)	193 (3.1)	
Other means	6 (2.9)	461 (7.6)	467 (7.4)	
**Time of injury**				
Morning (4 am to <12 noon)	56 (26.9)	1,518 (24.9)	1,574 (24.9)	0.3016
Afternoon (12 noon to <6 pm)	81 (38.9)	2,106 (34.5)	2,187 (34.6)	
Evening (6 pm to <10 pm)	62 (29.8)	2,190 (35.9)	2,252 (35.7)	
Late night (10 pm to <4 am)	9 (4.3)	292(4.8)	301 (4.8)	
**Day of week injury occurred**			
Sunday	23 (11.1)	894 (14.6)	917 (14.5)	0.0001
Monday	22 (10.6)	957 (15.7)	979 (15.5)	
Tuesday	36 (17.3)	797 (13.1)	833 (13.2)	
Wednesday	32 (15.4)	835 (13.7)	867 (13.7)	
Thursday	50 (24.0)	862 (14.1)	912 (14.4)	
Friday	18 (8.7)	897 (14.7)	915 (14.5)	
Saturday	27 (13.0)	864 (14.2)	891 (14.1)	
**Place of injury**			
Street	159 (76.4)	3,349 (54.9)	3,508 (55.6)	
Home	26 (12.5)	1,863 (30.5)	1,889 (30.0)	<0.001
Bar, or similar location of socializing	9 (4.3)	34 (0.6)	43 (0.7)	
Work	8 (3.9)	249 (4.1)	257 (4.1)	
School	3 (1.4)	135 (2.2)	138 (2.2)	
Market	1 (0.5)	19 (0.3)	20 (0.3)	
Other	1 (0.5)	456 (7.5)	457 (7.2)	
Unknown	1 (0.5)	1 (0.02)	2 (0.03)	
**How Injury was sustained**			
Transport	146 (70.2)	2,836 (46.5)	2,982 (47.2)	<0.001
Fall from height	15 (7.2)	417 (6.8)	432 (6.8)	
Contact with foreign body	9 (4.3)	190 (3.1)	199 (3.2)	
Domestic (relational-husband/wife/family)	7 (3.4)	199 (3.3)	206 (3.3)	
Street/compound fight	7 (3.4)	247 (4.1)	254 (4.0)	
Stab/cut	7 (3.4)	667 (10.9)	674 (10.7)	
Traveling	1 (0.5)	14 (0.2)	15 (0.2)	
Electricity	1 (0.5)	24 (0.4)	25 (0.4)	
Bite (person/dog/snake/other animal)	1 (0.5)	982 (16.1)	983 (15.6)	
Sexual assault	0 (0.0)	19 (0.3)	10 (0.3)	
Gunshot	0 (0.0)	27 (0.4)	27 (0.4)	
Choking/strangulation	0 (0.0)	17 (0.3)	17 (0.3)	
Drowning/near drowning	0 (0.0)	6 (0.10)	6 (0.10)	
Natural disaster	0 (0.0)	47 (0.8)	47 (0.7)	
Fire/smoke/heat (includes warm liquids and fireworks)	0 (0.0)	153 (2.5)	153 (2.4)	
Poisoning (includes drugs, pesticides, cooking fuel, detergent)	0 (0.0)	86 (1.4)	86 (1.4)	
Other	13 (6.3)	171 (2.8)	184 (2.9)	
Unknown	1 (0.5)	4 (0.1)	5 (0.1)	
**Kampala trauma score (KTS II) at time of presentation**			
	*n* = 159	*n* = 4,105	*n* = 4,264	
1 Severe (< =6)	26 (16.4)	63 (1.5)	89 (2.1)	<0.001
2 Moderate (7–8)	61 (38.4)	1,865 (45.4)	1,926 (45.2)	
3 Mild (9–10)	72 (45.3)	2,177 (53.0)	2,249 (52.7)	
**Outcomes at discharge**				
Alive and well	156 (93.2)	5,688 (75.0)	5,844 (92.6)	<0.001
Alive with disability	0 (0.0)	47 (0.8)	47 (0.7)	
Referred to other hospital	26 (12.5)	286 (4.7)	312 (4.9)	
Self-discharge	0 (0.0)	12 (0.2)	12 (0.2)	
Died	23 (11.1)	63 (1.0)	86 (1.4)	
Other	2 (1.0)	5 (0.1)	7 (0.1)	
Unknown	1 (0.5)	5 (0.1)	6 (0.1)	

To better understand the head-injured patients' outcomes (and possibly the care), we cross-tabulated the level of consciousness and injury severity with the patients' discharge dispositions in Table 3. Of the 177 majority who arrived conscious, 150 (84.8%) were discharged alive and well. However, the greatest morbidity and mortality burden was observed in the 26 patients who arrived unconscious at the hospital facilities. Eight (30.8%) were referred to a more equipped facility for higher-level care, 11 (42.3%) died, and only 6 of the 26 (23.1%) were discharged without event. Using the Kampala Trauma Score (KTS II) (22) to evaluate the association between injury severity at arrival and discharge status revealed that the majority of patients had mild to moderate KTS II scores. However, over a third of those with severe injuries (34.6%) were referred to more equipped facilities and over a quarter (26.9%) died.

**Table 3:** Level of consciousness at arrival, Kampala Trauma Score (KTS II) and discharge dispositions amongst patients with head injuries.

***n* (%)**	**Consciousness at arrival**		**Kampala Trauma Score (KTS II) at arrival**							
	**Alive and Conscious**	**Alive but Unconscious**	**Dead**	**Total**	***p*-value**	**Severe (** < = **6)**	**Moderate (7-8)**	**Mild (9–10)**	**Total**	***p*-value**
	*n* = 177 (85.1)	*n* = 26 (12.5)	*n* = 5 (2.4)	208	<0.001	*n* = 26 (16.4)	*n* = 61 (38.4)	*n* = 72 (45.3)	159	<0.001
**Outcomes at Discharge**				
Alive and well	150 (84.8)	6 (23.1)	-	156 (75.0)		9 (34.6)	53 (86.9)	66 (91.7)	128 (80.5)	
Alive with Disability	0 (0.0)	0 (0.0)	-	0 (0.0)		0 (0.0)	0 (0.0)	0 (0.0)	0 (0.0)	
Referred to other hospital	18 (10.2)	8 (30.8)	-	26 (12.5)		9 (34.6)	6 (9.8)	4 (5.6)	19 (12.0)	
Self-discharge	0 (0.0)	0 (0.0)	-	0 (0.0)		0 (0.0)	0 (0.0)	0 (0.0)	0 (0.0)	
Died	7 (4.0)	11 (42.3)	5 (100.0)	23 (11.1)		7 (26.9)	1 (1.6)	1 (1.4)	9 (5.7)	
Other	1 (0.6)	1 (3.9)	-	2 (0.96)		1 (3.9)	0 (0.0)	1 (1.4)	2 (1.3)	
Unknown	1 (0.6)	0 (0.0)	-	1 (0.48)		0 (0.0)	1 (1.6)	0 (0.0)	1 (0.6)	

**Figure 2:**
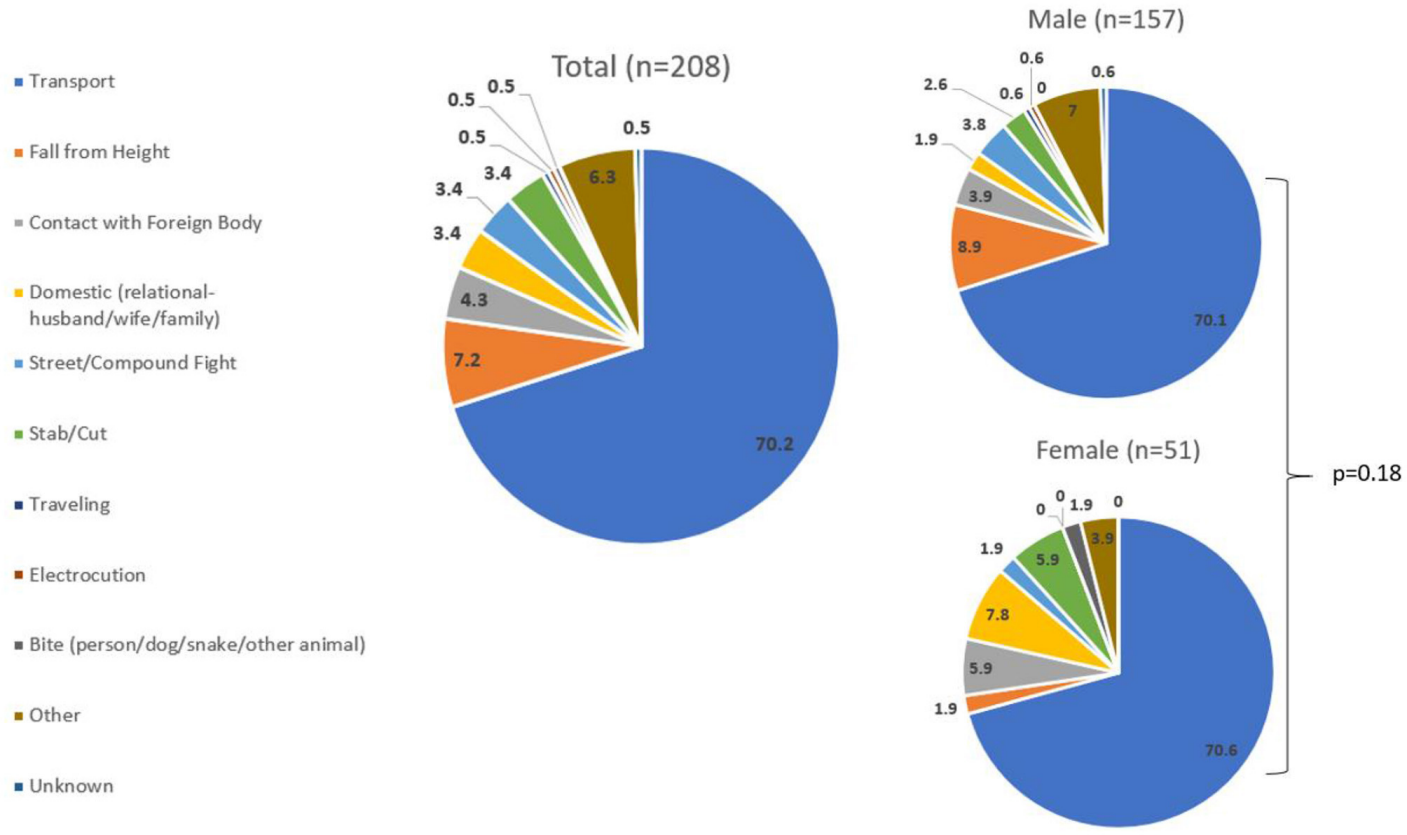
Mechanism of injury amongst head-injured patients, stratified by sex (%).

To fulfill the study's 2^nd^ objective, we examined sex differences in head injury outcomes for points of intervention. Figure 2 shows that regardless of sex, 70% of patients sustained their injuries in transport-related events. For males, the 2^nd^ most common mechanism was falls from height (8.9%), and for females, it was domestic violence (7.8%). When evaluating if sex predicts the odds of head injury (Table 4), age-adjusted models showed that males had 1.4 times the odds of head injuries compared to females (*p* = 0.03) and 2.7 times the odds of mortality from head injuries (*p* = 0.13); though the latter lacked statistical significance.

**Table 4:** Sex-predicted outcomes for patients in the Kintampo Registry.

**Variable**	**OR (95% CI)**	***p*-value**	**AOR (95% CI)**	***p*-value**
**Does sex predict the odds of head injury? (*****n*** **=** **6,314)**
Female	1		1	
Male	1.5 (1.08, 2.06)	0.0144	1.4 (1.04; 2.00)	0.0301
**Within the head-injured group, are there sex differences in mortality? (*****n*** **=** **208)**
Female	1		1	
Male	2.5 (0.69, 8.69)	0.1635	2.7 (0.74; 10.00)	0.1319

*OR, Crude odds ratio; AOR, Age-adjusted odds ratio; adjusted for age categories <19 years, 20–39 years (REF) and ≥40 years*.

## Discussion

According to the World Health Organization (WHO), approximately 85% of the world's population resides in low and middle-income countries (LMIC), where 90% of injuries occur (2, 8, 23). Yet injuries in these resource-limited health contexts are under-represented across the literature. The global incidence rate of HI is estimated at 200 per 100 000 people per year. However, as discussed earlier, this rate is grossly under-estimated due to the under-representation of cases in several LMICs. Despite this methodological limitation, it cannot be denied that many years of productive life are lost to HI each year and survivors are faced with years of disability and subsequent impairments in their quality of life. The economic ramifications of the injury on individuals, families, and society at large also cannot be overstated, particularly since the injury tends to affect people in the “prime” years of their lives. Understanding the HI prevalence in resource-limited settings such as Ghana presents opportunities for the creation of policies and interventions that have the potential to reduce the injury's impact on the individual and across all levels of community and society.

This study evaluated the HI prevalence in the Kintampo Injury Registry, a collaborative effort between Kintampo Health Research Centre (KHRC) in Ghana and the sidHARTe Program at Columbia University Mailman School of Public Health. The HI prevalence was 3.3% in our study population, which translates to a population burden of 3,294 head injuries per 100,000, a substantial increase above the global average. However, to most global HI researchers, this estimate may not be a surprise, as it is on par with other countries in the region, such as South Africa, which has an annual TBI rate that is 1.5 to 3.5 times that of the global estimate (8). LMICs carry not only the world's greatest burden of all injuries but also bear the global burden of head injuries. In assessing HI patients' utilizing of an academic neurosurgery unit in Nigeria, Adeleye et al. (24) found that 78% of their patients were managed non-operatively, 11.6% received operative care, and many other surgical candidates died before surgery or had no funding for this undertaking. In reviewing our findings, some critical questions come to mind: how were the patients triaged? What clinical interventions did they receive in-hospital? What was the financial impact of the injury, and who assumed those financial liabilities? The clinical interventions required by these head-injured patients, and their associated costs, are critical factors for stakeholders, policymakers and funders to consider as they strategize and prioritize the needs of patients in LMIC settings. As shown by Brouillette et al. (25), even if the annual case volume of Ghanaian and US trauma centers are equivalent, Ghanaian institutions tend to be under-resourced, under-staffed, and over-burdened with a disproportionately larger number of trauma cases, severe fractures, and infections compared to the more-resourced American facility. Equitable distribution of training resources and interventions should be on the agenda for stakeholders and funders as we move forward in the agenda to address TBI-related health disparities globally.

Our study's findings echo that of research teams in South Africa (8), Ethiopia (14), Rwanda (11), and Nigeria (13, 21, 24, 26), which show an increased and increasing burden of head injuries across the African continent and expose the vulnerability of Africa's growing populations to transport/road traffic-related head injuries. Rapid global expansion, infrastructure development, and increasing motorization across the African continent have collided with extensive deterioration of national road infrastructures, which has resulted in a daily deluge of road-traffic-related cases of HI in most clinical facilities (24, 26, 27). Ironically, after their injuries, most patients in our study were transported across the same, suboptimal road infrastructures to the hospital facilities *via* taxis/commercial vehicles rather than ambulances, threatening their chances for survival. Mode of arrival may not impact those with “mild” or moderate head injuries (who were often discharged without consequence). However, for those with more severe head injuries, ambulance transport could improve their chances for survival by reducing the “golden hour” (27, 28). In our study, a more significant proportion of head-injured patients arrived at the hospital dead or unconscious, and head-injured patients had 13 times the odds of mortality compared to those without head injuries (OR: 13.3; 95% CI: 8.05, 22.0; *p* < 0.0001) yet ambulance use was greater in the head-injured than the non-head injured group. Are ambulances well equipped to traverse the poor road infrastructures? What was the average time to hospital arrival? What interventions were administered to patients in the ambulances? Were head-injured patients dead on ambulance arrival? The answers to these questions require extensive mixed-methods assessments that will aid health disparities researchers and implementation scientists to better understand and improve HI outcomes for populations in LMICs.

Recently, the literature on “gender as a social determinant of health” has gained immense momentum, partly due to the COVID-19 pandemic, which shed light on the detrimental impact of gender norms on health equity and well-being (29). Our second aim sought to identify potential intervention points by looking at sex differences in HI outcomes. Our results showed that although there were no statistically significant differences in injury mechanism, males were more likely to sustain a head injury than females and males were more likely to die from their head injuries than their female peers. Why is there such a difference between the sexes? Why are men more likely to succumb to their injuries than women when their mechanisms of injury are mostly due to road traffic/transport-related accidents? In a highly gendered society like Ghana (30), where norms often dictate status, we must better understand the direct and indirect cultural, economic, and societal ramifications of these injuries on the victims. What happens to women and children when the male breadwinner dies or is disabled from an injury? Do survivors suffer any post-traumatic effects? How are their functional outcomes after injury? Longitudinal assessments are needed to better understand the long-term, out-of hospital consequences of HI in LMIC settings as being “discharged alive and well” may not correlate with quality of life outcomes (31). Research is also needed to better understand the role of domestic violence/disputes on women's long-term outcomes after HI, since Sub-Saharan Africa (SSA) has one of the highest rates of intimate partner violence (32).

## Strengths and Limitations

Our study's strengths lie in the large sample size of over 6,000 patients, which accommodated various statistical evaluations in these secondary data analyses. Our comprehensive approach to data collection also limited the frequency of missing data and reduced the need for imputations. Thus, the results reflect the actual responses of the study participants. The study, however, is limited in its generalizability to facilities across the entire country of Ghana, as the data was collected only the middle-belt region of the country.

## Conclusion

Head Injury is one of the leading causes of morbidity and mortality globally, and Ghanaians are no exception. Our results showed that HI was a significant risk for mortality; particularly amongst males. Data on the in-hospital procedures performed on the patients and longitudinal assessments of their long-term outcomes will better contextualize future research endeavors. This study provides the impetus for shaping prevention and treatment policy around HI prevention in LMIC's like Ghana.

## Data Availability Statement

Upon reasonable request, the raw data supporting the conclusions of this article will be made available by the authors, without undue reservation.

## Ethics Statement

The studies involving human participants were reviewed and approved by Institutional Ethics Committee of KHRC, the Institutional Review Board at Columbia University and authorities of the participating health facilities. Written informed consent to participate in this study was provided by the participants' legal guardian/next of kin.

## Author Contributions

GA: data curation and validation. MA-O and GA: formal analysis. FB, GA, SG, JA, DP, SO-A, and RM: project administration, resources, and supervision. FB and MA-O: writing—original draft. All authors: conceptualization, investigation, methodology, and writing—review and editing. All authors contributed to the article and approved the submitted version.

## Funding

This work was supported by The GE Foundation and The sidHARTe-Strengthening Emergency Systems Program at Columbia University Mailman School of Public Health and. At the time of press, MA-O was funded by NIH NINDS K01 Award# 5K01NS121199-03.

## Conflict of Interest

The authors declare that the research was conducted in the absence of any commercial or financial relationships that could be construed as a potential conflict of interest.

## Publisher's Note

All claims expressed in this article are solely those of the authors and do not necessarily represent those of their affiliated organizations, or those of the publisher, the editors and the reviewers. Any product that may be evaluated in this article, or claim that may be made by its manufacturer, is not guaranteed or endorsed by the publisher.

## References

[1] Sharma B Lawrence DW. Top-cited articles in traumatic brain injury. Front Hum Neurosci. (2014) 8:879. 10.3389/fnhum.2014.00879 25414657 PMC4220681

[2] Gosselin RA Spiegel DA Coughlin R Zirkle LG. Injuries: the neglected burden in developing countries. Bull World Health Organ. (2009) 87:246–246a. 10.2471/BLT.08.052290 19551225 PMC2672580

[3] Global regional and and national burden of traumatic brain injury and spinal cord injury 1990-2016: 1990-2016: a systematic analysis for the Global Burden of Disease Study 2016. Lancet Neurol. (2019) 18:56–87. 30497965 10.1016/S1474-4422(18)30415-0 PMC6291456

[4] Johns Hopkins Medicine,. Head injury (2022). Available online at: https://www.hopkinsmedicine.org/health/conditions-and-diseases/head-injury (accessed March 22, 2022).

[5] Zitnay GA Zitnay KM Povlishock JT Hall ED. Traumatic brain injury research priorities: the Conemaugh International Brain Injury Symposium. J Neurotrauma. (2008) 25:1135–52. 10.1089/neu.2008.0599 18842105

[6] Nguyen R Fiest KM McChesney J Kwon CS Jette N Frolkis AD. The International Incidence of Traumatic Brain Injury: A Systematic Review and Meta-Analysis. Can J Neurol Sci. (2016) 43:774–85. 10.1017/cjn.2016.290 27670907

[7] Nguyen R Fiest K Jette N Gallagher C. Response to “Review of the Incidence of Traumatic Brain Injury”. Can J Neurol Sci. (2017) 44:612. 10.1017/cjn.2017.198 28735595

[8] Bryan-Hancock C Harrison J. The global burden of traumatic brain injury: preliminary results from the Global Burden of Disease Project. Inj Prev. (2010) 16(Suppl 1):A17. 10.1136/ip.2010.029215.61

[9] Seidenberg P Cerwensky K Brown RO Hammond E Mofu Y Lungu J. Epidemiology of injuries, outcomes, and hospital resource utilisation at a tertiary teaching hospital in Lusaka, Zambia. Afr J Emerg Med. (2014) 4:115–22. 10.1016/j.afjem.2014.01.006

[10] Chichom Mefire A Etoundi Mballa GA Azabji Kenfack M Juillard C Stevens K. Hospital-based injury data from level III institution in Cameroon: retrospective analysis of the present registration system. Injury. (2013) 44:139–43. 10.1016/j.injury.2011.10.026 22098714

[11] Mbanjumucyo G George N Kearney A Karim N Aluisio AR Mutabazi Z. Epidemiology of injuries and outcomes among trauma patients receiving prehospital care at a tertiary teaching hospital in Kigali, Rwanda. Afr J Emerg Med. (2016) 6:191–7. 10.1016/j.afjem.2016.10.001 30456094 PMC6234177

[12] Samuel JC Akinkuotu A Villaveces A Charles AG Lee CN Hoffman IF. Epidemiology of injuries at a tertiary care center in Malawi. World J Surg. (2009) 33:1836–41. 10.1007/s00268-009-0113-4 19597877 PMC3290404

[13] Thanni LO Kehinde OA. Trauma at a Nigerian teaching hospital: pattern and docu-mentation of presentation. Afr Health Sci. (2006) 6:104–7. 16916301 10.5555/afhs.2006.6.2.104 PMC1831976

[14] Taye M Munie T. Trauma registry in Tikur Anbessa Hospital, Addis Ababa, Ethiopia. Ethiop Med J. (2003) 41:221–6. 15227887

[15] Bhalla K Adofo K Mock CN Afukaar F Appiah N Ebel BE. Non-traditional data sources for injury control: an agenda for action in Ghana. Injury Prevention. (2012) 18:277. 10.1136/injuryprev-2012-040410 22729163

[16] Mock CN Abantanga F Cummings P Koepsell TD. Incidence and outcome of injury in Ghana: a community-based survey. Bull World Health Organ. (1999) 77:955–64. 10680242 PMC2557773

[17] Mock CN Adzotor E Denno D Conklin E Rivara F. Admissions for injury at a rural hospital in Ghana: implications for prevention in the developing world. Am J Public Health. (1995) 85:927–31. 10.2105/AJPH.85.7.927 7604915 PMC1615531

[18] Ghana Statistical Service. Ghana 2021 Population Housing Census. (2021). Available online at: http://census2021.statsghana.gov.gh/ (accessed March 30, 2022).

[19] The World Bank. Rising through Cities in Ghana: The time for action is now to fully benefit from the gains of urbanization. (2015). Available online at: https://www.worldbank.org/en/news/opinion/2015/05/14/rising-through-cities-in-ghana-the-time-for-action-is-now-to-fully-benefit-from-the-gains-of-urbanization (accessed March 28, 2022).

[20] World Health Organization. International Statistical Classification of Diseases and Related Health Problems. (2011). Available online at: https://www.who.int/standards/classifications/classification-of-diseases (accessed January 20, 2022).

[21] Zavalaa DE Bokongo S John IA Senoga IM Mtonga RE Mohammed AZ. Implementing a hospital based injury surveillance system in Africa: lessons learned. Med Confl Surviv. (2008) 24:260–72. 10.1080/13623690802373884 19065866

[22] Weeks SR Juillard CJ Monono ME Etoundi GA Ngamby MK Hyder AA. Is the Kampala trauma score an effective predictor of mortality in low-resource settings? A comparison of multiple trauma severity scores. World J Surg. (2014) 38:1905–11. 10.1007/s00268-014-2496-0 24715042

[23] Hofman K Primack A Keusch G Hrynkow S. Addressing the growing burden of trauma and injury in low- and middle-income countries. Am J Public Health. (2005) 95:13–7. 10.2105/AJPH.2004.039354 15623852 PMC1449844

[24] Adeleye AO Ogun MI. Clinical epidemiology of head injury from road-traffic trauma in a developing country in the current era. Front Neurol. (2017) 8:695. 10.3389/fneur.2017.00695 29326652 PMC5736536

[25] Brouillette MA Kaiser SP Konadu P Kumah-Ametepey RA Aidoo AJ Coughlin RC. Orthopedic surgery in the developing world: workforce and operative volumes in Ghana compared to those in the United States. World J Surg. (2014) 38:849–57. 10.1007/s00268-013-2314-0 24218152

[26] Adeleye AO Clark DJ Malomo TA. Trauma demography and clinical epidemiology of motorcycle crash-related head injury in a neurosurgery practice in an African developing country. Traffic Inj Prev. (2019) 20:211–5. 10.1080/15389588.2018.1553085 30946601

[27] Karthigeyan M Gupta SK Salunke P Dhandapani S Wankhede LS Kumar A. Head injury care in a low- and middle-income country tertiary trauma center: epidemiology, systemic lacunae, possible leads. Acta Neurochir. (2021) 163:2919–30. 10.1007/s00701-021-04908-x 34159448

[28] Dinh MM Bein K Roncal S Byrne CM Petchell J Brennan J. Redefining the golden hour for severe head injury in an urban setting: the effect of prehospital arrival times on patient outcomes. Injury. (2013) 44:606–10. 10.1016/j.injury.2012.01.011 22336130

[29] Lungumbu S Butterly A BBC, News,. Coronavirus gender: More chores for women set back gains in equality. (2020). Available online at: https://www.bbc.com/news/world-55016842 (accessed February 15, 2022).

[30] Wahabu E Patel P. Rural women are reshaping gender norms in northern Ghana. (2020). Available online at: https://www.iwmi.cgiar.org/2020/10/rural-women-are-reshaping-gender-norms-in-northern-ghana/ (accessed March 21, 2022).

[31] Ding K Sur PJ Mbianyor MA Carvalho M Oke R Dissak-Delon FN. Mobile telephone follow-up assessment of postdischarge death and disability due to trauma in Cameroon: a prospective cohort study. BMJ Open. (2022) 12:e056433. 10.1136/bmjopen-2021-056433 35383070 PMC8984008

[32] World Health Organization. Violence against women prevalence estimates, 2018–Executive summary. (2018). Available online at: https://www.who.int/publications/i/item/9789240026681 (accessed March 28, 2022).

